# Editorial: The relationship of neuroinflammation with aging and neurodegenerative diseases

**DOI:** 10.3389/fnagi.2022.1102613

**Published:** 2022-12-09

**Authors:** Guohao Wang, Peng Yin, Wei Zhang, Celia Giulietta Fernandez, Xingshun Xu

**Affiliations:** ^1^Synapse and Neural Circuit Section, National Institute of Neurological Disorders and Stroke, National Institutes of Health, Bethesda, MD, United States; ^2^Guangdong Key Laboratory of Non-human Primate Research, Guangdong-Hongkong-Macau Institute of CNS Regeneration, Jinan University, Guangzhou, China; ^3^Neural Stem Cell Research Lab, Research Department, National Neuroscience Institute, Singapore, Singapore; ^4^The Neurodegeneration Consortium, Institute of Applied Cancer Science (IACS), The University of Texas MD Anderson Cancer Center, Houston, TX, United States; ^5^Department of Neurology, The First Affiliated Hospital of Soochow University, Suzhou, China; ^6^Institute of Neuroscience, Soochow University, Suzhou, China; ^7^Jiangsu Key Laboratory of Neuropsychiatric Diseases, Soochow University, Suzhou, Jiangsu, China

**Keywords:** neuroinflammation, neurodegenerative diseases, NLRP3 inflammasome, phosphatidylserine (PS), C-reactive protein

Neuroinflammation exists in variety of aging-related neurodegenerative diseases, including Alzheimer's disease (AD), Parkinson's disease (PD), Huntington's disease (HD), amyotrophic lateral sclerosis (ALS), major depressive disorder (MDD), ischemic stroke, spinal cord injury, and schizophrenia (Colonna and Brioschi, 2020). Among the many critical molecules that regulate neuroinflammation, the NLRP3 (NOD-, LRR- and pyrin domain-containing protein 3) inflammasome complex was found to play important roles in cellular immune response such as during stress and infection. Assembly of the activated NLRP3 inflammasome leads to the cleavage of pro-Caspase-1 to Caspase-1. Caspase-1 releases cytokines interleukin-1β and interleukin-18, promoting Gasdermin D-mediated, pyroptotic cell death (Swanson et al., 2019; Wang et al., 2021). Recent evidence demonstrates that NLRP3-medicated neuroinflammation is involved in the pathology of neurodegenerative diseases. Misfolded protein aggregates in neurons are well-known, cellular hallmarks in a variety of neurodegenerative diseases.

Misfolded proteins and damaged organelles are degraded by inflammation-related cells such as microglia in the brain. Activated-NLRP3 inflammasome in microglia plays a key role for fighting with misfolded proteins to rescue neurons. Autophagy and ubiquitin-proteasome system in neurons also take part in the degradation or recycling of these mutant aggregates proteins. However, excessive aggregates in neurons impair the autophagy and ubiquitin protein degradation system, leading to activation of NLRP3 inflammasomes in microglia and neuronal death. Microglia activates NLRP3 inflammasomes and releases cytokines in response to toxic protein aggregates in neurodegenerative diseases.

AD is a common, age-dependent neurodegenerative disease which is characterized with the accumulation of amyloid beta (Aβ) and intracellular neurofibrillary tangles composed of hyperphosphorylated tau protein aggregates. The Aβ polypeptide aggregates in brain are considered as a key pathological hallmark of AD. Interestingly, NLRP3 inflammasomes were found in AD patient's brain and animal models (Lu et al.). Thus, approaches to degrading protein aggregates by the autophagy system and inhibit the neuroinflammation is a promising direction for the treatment of neurodegenerative diseases characterized by misfolded proteins.

Interestingly, oxygen atom attachment to Aβ and Tau by using photo catalysts reduces their toxicity in neurons by suppressing aggregate formation. The β-sheet structure is the target for ligate a small compound by photo-oxygenation reaction. This is activated by light irradiation and then generated singlet oxygenation when they relax to their basic state which results in the selective photo-oxygenated Aβ and tau proteins. In addition, this method also activates microglia to clear Aβ and tau proteins. Although this photo-oxygenation method provides a new strategy for the clearance of misfolded Aβ and tau proteins, it can be used as a treatment strategy for other misfolded protein diseases, including PD, ALS, and HD (Tomizawa et al.).

In addition to photo-oxygenation, immunotherapy may be an important approach to clear misfolded Aβ and tau proteins. Immunotherapy enhances autophagic processes in microglia and is a relatively new approach for clearing Aβ aggregates. The concept of AD immunotherapy originates from cancer immunotherapy, in which injection of soluble Aβ fragments and tau proteins produces anti-Aβ and anti-tau antibodies. An increase in microglial phagocytosis of these aggregates and a reduction of Aβ plaques has been observed following injections of Aβ into AD patients (Bard et al., 2000; Nicoll et al., 2019). The United States Food and Drug Administration (FDA) has approved aducanumab, a human monoclonal antibody that recognizes aggregated Aβ, for the treatment of AD. Indeed, it is a big step for treatment of neurodegenerative disease through immunotherapy (Sevigny et al., 2016).

Phosphatidylserine (PS) is a membrane component that accounts for 5–10% of total lipids in cells, which connects many signals to be involved in a variety of biological process, such as apoptosis, signaling transmission, inflammation, synapse refinement, and enzyme activation in neurons. Dysregulation of PS was found in many neurodegenerative diseases and psychiatry diseases (Ma et al.). Supplementation of PS to patients with AD and PD has been shown to alleviate age-related, cognitive impairment. Importantly, exogenous PS liposomes exhibit anti-neuroinflammation effects in the brain by regulating microglial cytokine release. It is observed that serum PS levels are changed in various neurodegenerative diseases and increasing PS level in the peripheral blood of MDD patients can improve the depression behaviors through the intervention of cytokine release such as tumor necrosis factor (TNF) release. Therefore, PS as a food supplementation can be used in the patients with AD, PD, or MDD.

Interestingly, TNF is also found to be associated with hearing loss. Specifically, serum TNF levels significant increases in patients with hearing loss. TNF levels have also been shown to increases with aging in human and DBA/2J mice. It has also been shown that low concentrations of TNF activate the NF-κB pathway to promote cell survival. Alternatively, high concentrations of TNF activate Caspase-3 to induce apoptosis of hearing cells, indicating that inflammation is also involved the mechisms of hearing loss (Wu et al.).

Shortened leukocyte telomere length (LTL) is also a known biomarker for aging and age-related diseases. A previous study demonstrated that smoking-produced, serum cotinine is associated with LTL and modulated by body mass index (BMI) and C-reactive protein (CRP). In total, about 4,047 adults were recruited in this study during 1999 to 2002 by a National Health and Nutrition Examination Survey. There was a significant, negative correlation of serum cotinine with LTL when BMI and CRP were excluded. When BMI and CRP were considered, the correlation of serum cotinine with LTL still remained significantly negative. However, BMI and CRP had significant incremental and mitigated effects on the negative association between the cotinine and LTL. Thus, this study indicates that inflammation and body weight have different roles on the correlation between serum cotinine and LTL. Therefore, smoking-associated BMI and inflammation may affect biological aging as represented by LTL (Gao et al.).

Altogether, these findings demonstrate that excess inflammation is considered one of the major culprits for neurodegenerative diseases, such as in AD, PD, and hearing loss. During inflammation, microglia activation plays a critical role in modulating neuronal function and cell death. Since microglia activation appears to be a “double-edged sword” that may contribute to neurodegenerative process, proper balance and maintenance of microglia activation may be an important strategy in the treatment of degenerative diseases and requires further investigation.

## Author contributions

GW drafted the manuscript. XX help to revise the full manuscript. All authors contributed to the article and approved the submitted version.

## References

[B1] Bard F. Cannon C. Barbour R. Burke R.-L. Games D. Grajeda H.. (2000). Peripherally administered antibodies against amyloid β-peptide enter the central nervous system and reduce pathology in a mouse model of Alzheimer disease. Nat. Med. 6, 916–919. 10.1038/78682 10932230

[B2] Colonna M. Brioschi S. (2020). Neuroinflammation and neurodegeneration in human brain at single-cell resolution. Nat. Rev. Immunol. 20, 81–82. 10.1038/s41577-019-0262-0 31844328

[B3] Nicoll J. A. R. Buckland G. R. Harrison C. H. Page A. Harris S. Love S.. (2019). Persistent neuropathological effects 14 years following amyloid-β immunization in Alzheimer's disease. Brain 142, 2113–2126. 10.1093/brain/awz142 31157360 PMC6598630

[B4] Sevigny J. Chiao P. Bussière T. Weinreb P. H. Williams L. Maier M.. (2016). The antibody aducanumab reduces Aβ plaques in Alzheimer's disease. Nature 537, 50–56. 10.1038/nature19323 27582220

[B5] Swanson K. V. Deng M. Ting J. P. (2019). The NLRP3 inflammasome: molecular activation and regulation to therapeutics. Nat. Rev. Immunol. 19, 477–489. 10.1038/s41577-019-0165-0 31036962 PMC7807242

[B6] Wang G. Yin W. Shin H. Tian Q. Lu W. Hou X. S. (2021). Neuronal accumulation of peroxidated lipids promotes demyelination and neurodegeneration through the activation of the microglial NLRP3 inflammasome. Nat. Aging. 1, 1024–1037. 10.1038/s43587-021-00130-7 37118341

